# Postural stability and plantar pressure parameters in healthy subjects: variability, correlation analysis and differences under open and closed eye conditions

**DOI:** 10.3389/fbioe.2023.1198120

**Published:** 2023-07-20

**Authors:** P. De Blasiis, P. Caravaggi, A. Fullin, A. Leardini, A. Lucariello, A. Perna, G. Guerra, A. De Luca

**Affiliations:** ^1^ Department of Mental and Physical Health and Preventive Medicine, Section of Human Anatomy, University of Campania “Luigi Vanvitelli”, Naples, Italy; ^2^ Movement Analysis Laboratory, IRCCS Istituto Ortopedico Rizzoli, Bologna, Italy; ^3^ Department of Advanced Biomedical Sciences, University of Naples “Federico II”, Naples, Italy; ^4^ Department of Sport Sciences and Wellness, University of Naples “Parthenope”, Naples, Italy; ^5^ Department of Medicine and Health Sciences “Vincenzo Tiberio”, University of Molise, Campobasso, Italy

**Keywords:** posture, postural control, foot, baropodometry, stabilometry, pressure plate, visual control

## Abstract

**Introduction:** The “postural control system” acts through biomechanical strategies and functional neuromuscular adaptations to maintain body balance under static and dynamic conditions. Postural stability and body weight distribution can be affected by external sensory inputs, such as different visual stimuli. Little information is available about the influence of visual receptors on stabilometric and plantar pressure parameters. The aim of this study was to analyze variability, correlations, and changes in these parameters under open- (OE) and closed-eye (CE) conditions.

**Methods:** A total of 31 stabilometric and plantar pressure parameters were acquired in 20 young and healthy adults during baropodometric examination performed in bipedal standing under both visual conditions. Variability of parameters was evaluated via the coefficient of variation, correlation analysis via Pearson’s R2, and statistical differences via the Wilcoxon test.

**Results:** High intra-subject repeatability was found for all plantar pressure parameters and CoP-speed (CV < 40%) under OE and CE conditions, while CoP-sway area (CoPsa) and length surface function (LSF) showed larger variability (CV > 50%). Mean and peak pressures at midfoot and total foot loads showed the least number of significant correlations with other parameters under both visual conditions, whereas the arch-index and rearfoot loads showed the largest number of significant correlations. The limb side significantly affected most plantar pressure parameters. A trend of larger LSF and lower CoPsa and mean and peak pressures at the right forefoot was found under the CE condition.

**Discussion:** The present study provides a deeper insight into the associations between postural stability and foot load. Interesting postural adaptations, particularly with respect to different visual stimuli, the effect of the dominant side, and the specific role of the midfoot in balance control were highlighted.

## 1 Introduction

Postural stability represents the ability of the “postural control system” (PCS) (Takakusaki, 2017) in maintaining the vertical projection of the body center of mass (COM) within the feet contact area (Patti et al., 2018). This function of the central nervous system relies on biomechanical strategies and neuromuscular adaptation to stabilize the position of the body segments against the force of gravity (Ivanenko and Gurfinkel, 2018) and to maintain balance (Moffa et al., 2020) during different motor tasks. Bipedal standing is characterized by postural parameters in the sagittal plane (De Blasiis et al., 2021a) resulting from the synergic actions of anti-gravity muscles on the human skeleton to ensure minimal COM oscillations and low energy expenditure (Gagey et al., 1998).

Body oscillations can be quantitatively assessed by baropodometry using stabilometric tests (Rosário, 2014; Baumfeld et al., 2017). Several plantar-pressure-based measures have been proposed to evaluate postural stability: center of pressure (CoP), CoP-sway area (CoPsa), CoP-speed, and length surface function (LSF) (Fullin et al., 2022) (Table 1). In addition, the morphology of the foot contact area and body weight distribution across different plantar regions can be analyzed under static and dynamic conditions (Rosário, 2014; Fullin et al., 2022) using plantar pressure parameters (percentages of body weight distribution, mean and peak pressures, and arch index).

**TABLE 1:** Description and legend of plantar pressure and stabilometric parameters. Forefoot (Ff); midfoot (Mf); rearfoot (Rf); mean pressure (Pmean); maximum pressure (Pmax); center-of-pressure speed (CoP-speed); center-of-pressure sway area (CoPsa); length surface function (LSF); arch index (AI); and foot contact area (FCA).

Legend	Plantar pressure and stabilometric parameters		Description
1	Load Tf [%]	Left	Percentage of body weight distribution
2	Load Tf [%]	Right	
3	Load Rf [%]	Left	Percentage of body weight distribution at the rearfoot
4	Load Rf [%]	Right	
5	Load Mf [%]	Left	Percentage of body weight distribution at the midfoot
6	Load Mf [%]	Right	
7	Load Ff [%]	Left	Percentage of body weight distribution at the forefoot
8	Load Ff [%]	Right	
9	Pmean Tf [KPa]	Left	Mean pressure at the total foot
10	Pmean Tf [KPa]	Right	
11	Pmean Rf [KPa]	Left	Mean pressure at the rearfoot
12	Pmean Rf [KPa]	Right	
13	Pmean Mf [KPa]	Left	Mean pressure at the midfoot
14	Pmean Mf [KPa]	Right	
15	Pmean Ff [KPa]	Left	Mean pressure at the forefoot
16	Pmean Ff [KPa]	Right	
17	Pmax Tf [KPa]	Left	Peak pressure at the total foot
18	Pmax Tf [KPa]	Right	
19	Pmax Rf [KPa]	Left	Peak pressure at the rearfoot
20	Pmax Rf [KPa]	Right	
21	Pmax Mf [KPa]	Left	Peak pressure at the midfoot
22	Pmax Mf [KPa]	Right	
23	Pmax Ff [KPa]	Left	Peak pressure at the forefoot
24	Pmax Ff [KPa]	Right	
25	FCA [mm^2^]	Left	Foot contact area
26	FCA [mm^2^]	Right	
27	AI [%]	Left	Ratio between the midfoot contact area and foot contact area (without toes)
28	AI [%]	Right	
29	CoPsa [mm^2^]		Area of an ellipse containing the trajectory of the center of pressure
30	CoP-speed [mm/s]		Average velocity of the center of pressure
31	LSF [mm^-1^]		Ratio between the distance covered by the center of pressure (CoP-length) and CoP sway area (CoPsa)

All baropodometric parameters are affected by the continuous postural adaptations performed by the PCS, which integrates sensory inputs (somatosensory, visual, and vestibular) and elaborates motor outputs stabilizing the whole-body posture (Gagey et al., 1998). In particular, the contribution of the visual receptor to postural control has been investigated for its impact across different scientific fields (Stoffregen et al., 2000; Bellizzi et al., 2011; Nishiike et al., 2013). Maintaining balance control in vision-loss conditions is supported mainly by the vestibular and proprioceptive receptors, through postural adjustments that may be assessed quantitatively and instrumentally.

Quantifying the physiological variation of stabilometric and plantar pressure parameters in a normal population is critical to define the reference values to assess pathological conditions and identify the most reliable parameters to be used in the clinical setting and research. The intra-subject and intra-day variability in stabilometric and plantar pressure parameters have been reported in healthy subjects under open- (OE) (Fullin et al., 2022) and closed-eye (CE) conditions (Samson and Crowe, 1996; Gagey and Weber, 2010; Hébert-Losier and Murray, 2020). The inter-subject variability in a young healthy population in OE during the same day (Fullin et al., 2022), two sessions 1 week apart (Alves and Porfirio Borel, 2018), and intra-session and inter-session over 2 weeks (Baldini et al., 2013) has also been assessed. Several studies defined the standard reference values of the aforementioned parameters under OE conditions (Ohlendorf et al., 2019; Fullin et al., 2022) and under static OE and CE conditions (Prieto et al., 1996; Tanaka et al., 2000; Hue et al., 2006; Vieira Tde et al., 2009; Bellizzi et al., 2011; Baldini et al., 2013; Santana Castro et al., 2021; Nishiike et al., 2013; Rodríguez-Rubio et al., 2020; De Blasiis et al., 2021b; Quialheiro et al., 2021; Scoppa and Gallamini, 2021), reporting greater CoPsa, CoP-speed, and CoP-length in CE.

While several stabilometric parameters are affected by visual stimuli, little information is available on which baropodometric parameters characterize the different visual conditions. The aim of this study was to evaluate the effect of visual stimuli on postural stability and plantar pressure parameters in a young and healthy population and to analyze variability, correlations, and changes in these parameters under OE and CE conditions.

## 2 Materials and methods

**FIGURE 1:**
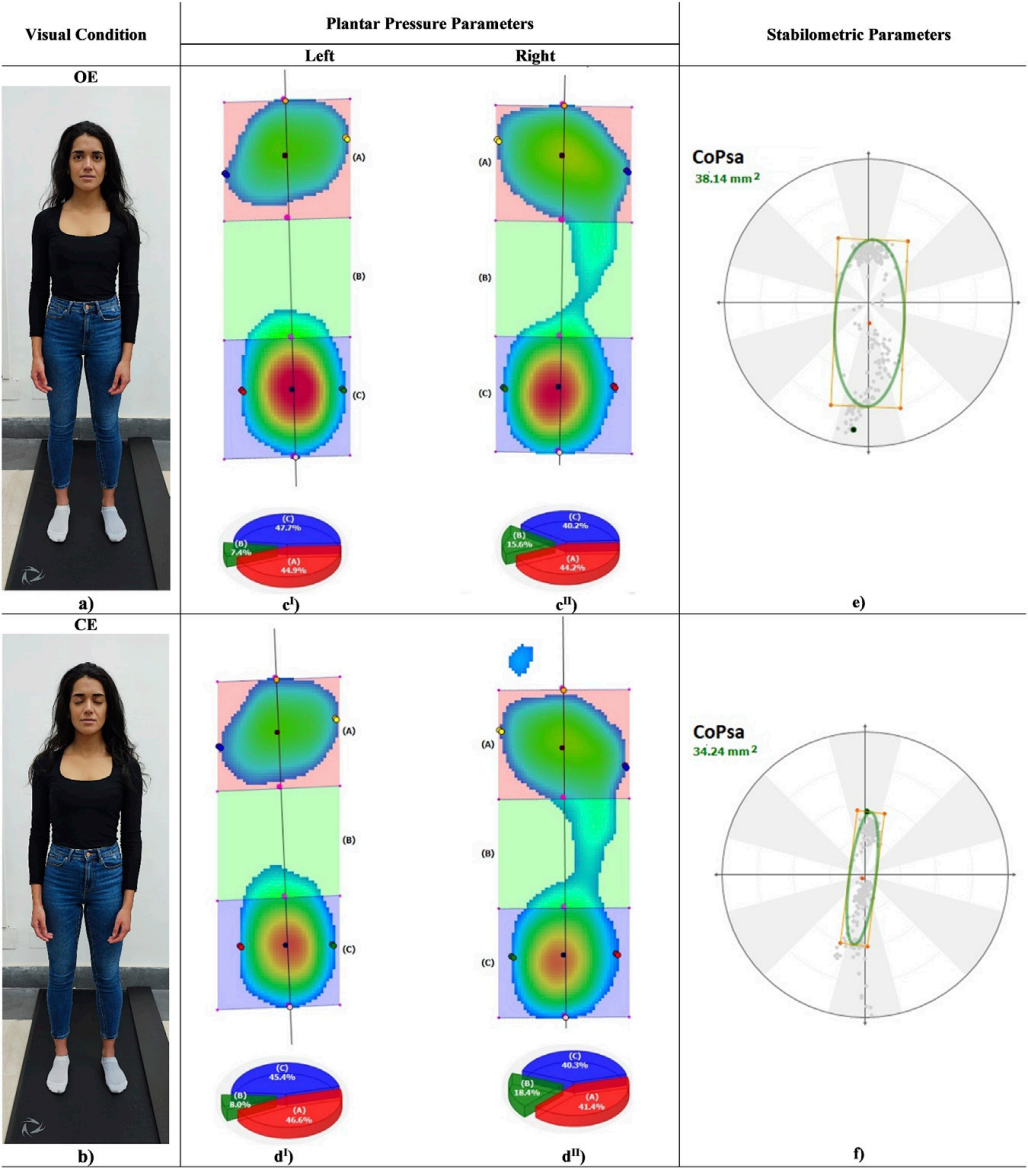
Left, a subject in anatomical standing posture in OE **(a)** and CE **(b)** conditions. Centre, color maps of plantar pressure distribution at rearfoot, midfoot and forefoot during standing in OE **(c^I^, c^II^)** and CE **(d^I^, d^II^)**. Right, center of pressure sway area (CoPsa) in OE **(e)** and CE **(f)**.

A total of 20 healthy subjects (7 M, 13 F; age = 20.2 ± 0.9 years; right-limb dominance = 20/20; height = 1.69 ± 0.09 m; weight = 61.94 ± 8.58 kg; BMI = 21.6 ± 1.7 kg/m^2^) were recruited at the Motion Analysis Laboratory of the Anatomy Department at the University of Campania L.Vanvitelli, Naples, Italy. Ethical review and approval were waived for this study due to the nature of this pilot study, which required the recruitment of a small population of healthy participants tested for standard plantar pressure parameters during bipedal standing posture. The following inclusion criteria were used: absence of pain, no surgery in the last 6 months, no muscle-skeletal injury in the last 3 months, no dental surgery or use of dental implants, no prostheses or use of corrective orthoses, no neurological or visual disease, no skeletal dysmorphism, and no cognitive impairment. Participants were evaluated in an anatomical upright bipedal posture with the arms relaxed along the body close to the thighs and the head in the neutral position using a 200 × 50 cm 10,000 sensors/m^2^ pressure plate (P-Walk FM12050 BTS-Bioengineering, Milan, Italy), sampling at 50 Hz (Figures 1A, B), following the international standardization criteria for baropodometric tests (Scoppa and Gallamini, 2017). The exceptions were for the visual target, placed 2.8 m away from the subject, and for the distance between the feet, self-selected by each participant with the indication to place the feet close but not together and to find a comfortable posture (Tarantola et al., 1997; Chiari et al., 2002). Four stabilometric exams of 30 s were performed on each participant under OE and CE conditions (Figure 1A, B). Each participant was allowed to sit down and rest before each trial, maintaining the same feet position in all the trials (Chiari et al., 2002). To ensure a correct postural examination, the tests were performed in silence and in a room with a level floor and white walls. The pressure plate was weight-calibrated before each measurement following the procedure recommended by the manufacturer. The following clinically relevant stabilometric and plantar pressure parameters were measured: center-of-pressure sway area (CoPsa; mm^2^); length surface function (LSF; mm^-1^); center-of-pressure speed (CoP-speed; mm/sec); total foot (Tf), rearfoot (Rf), midfoot (Mf), and forefoot (Ff) loads (%); mean and peak pressures (Pmean and Pmax; KPa) at Rf, Mf, and Ff; the foot contact area (FCA; mm^2^); and arch index (AI; %) (Figure 1C–F; Table 1) (Fullin et al., 2022). All plantar pressure parameters were calculated for both left (l) and right (r) sides. Mean pressure parameters at Rf, Mf, and Ff were calculated as the ratio between weight and area calculated for each region. Load parameters were normalized to body weight (%BW). For each parameter, intra-subject (inter-trial) variability was assessed via the coefficient of variation (CV) across four trials for each subject. Possible correlations between pairs of stabilometric and plantar pressure parameters were assessed under OE and CE conditions via Pearson’s correlation analysis (*R* ^2^). The effects of the side and the visual conditions were tested via the non-parametric Wilcoxon paired test. A Bonferroni correction was applied to the significance level (adjusted α = 0.005) to account for the multiple correlation analyses and for the multiple paired comparisons between OE and CE conditions. Statistical analysis was performed using MATLAB (MathWorks) and R (R Core Team. R, 2018).

## 3 Results

### 3.1 Variability in stabilometric and plantar pressure parameters under OE and CE conditions

**FIGURE 2:**
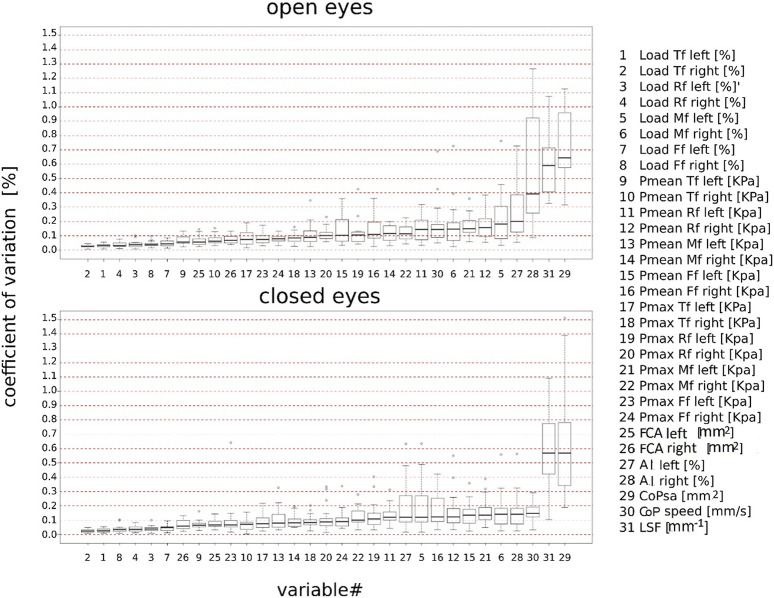
Boxplot of the intra-subject coefficient of variation (%) of stabilometric and plantar pressure parameters sorted in ascending order according to the median values under open- and closed-eye conditions. Total foot (Tf); forefoot (Ff); midfoot (Mf); rearfoot (Rf); mean pressure (Pmean); maximum pressure (Pmax); center-of-pressure speed (CoP-speed); length surface function (LSF); center-of-pressure sway area (CoPsa); arch index (AI); and foot contact area (FCA).

Figure 2 depicts the boxplots (median 25%–75%) for each parameter of the CV distribution across subjects. The CVs were sorted in ascending order along the *x*-axis by the median value. High repeatability (median CV < 20%) was observed for all plantar pressure parameters and CoP-speed under both visual conditions, except for right AI in OE (median CV = ∼40%). CoPsa and LSF showed the largest variability (median CV > 50%).

### 3.2 Correlation analysis across stabilometric and plantar pressure parameters under OE and CE conditions

**FIGURE 3:**
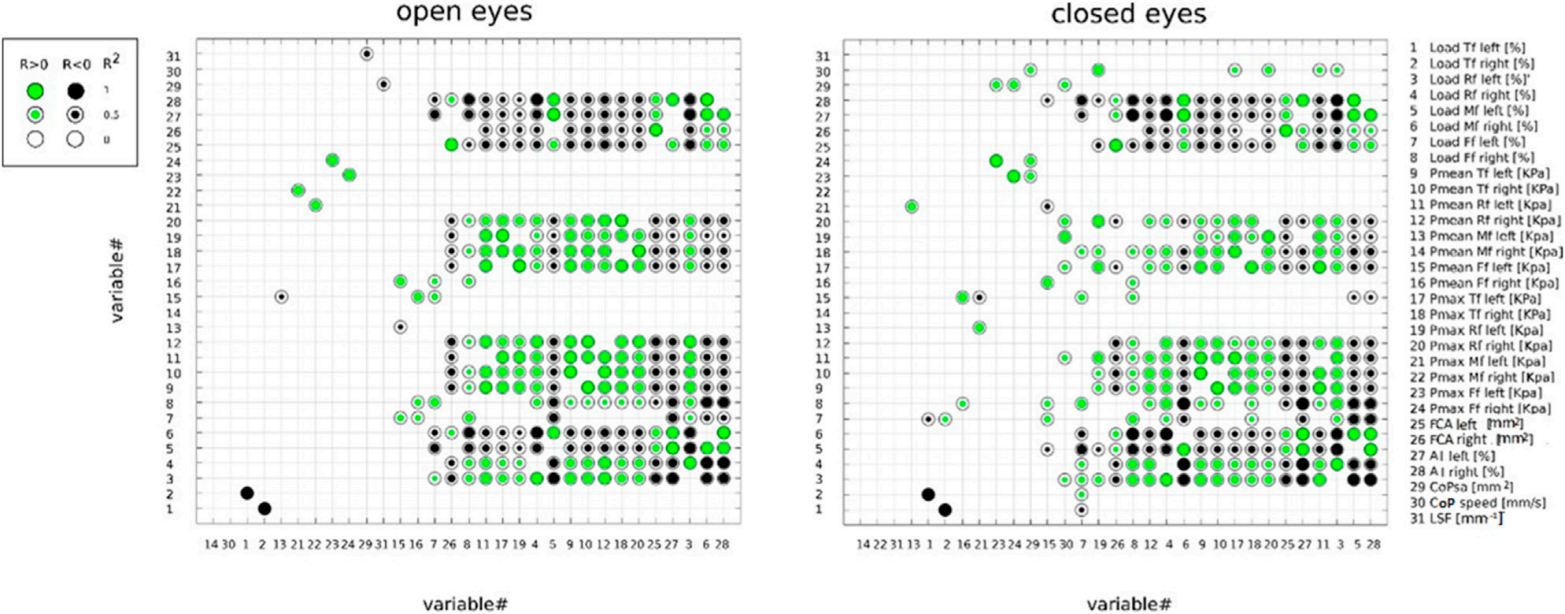
Correlation between pairs of stabilometric and plantar pressure parameters, assessed under open- and closed-eye conditions by Pearson’s correlation analysis (R^2^). Positive and negative correlations are represented by green and black round markers, respectively. The parameters were ordered from left to right on the *X*-axis in ascending order according to the number of statistically significant correlations with other parameters. The α level of significance was corrected to 0.005 accounting for the multiple correlations. Total foot (Tf); forefoot (Ff); midfoot (Mf); rearfoot (Rf); mean pressure (Pmean); maximum pressure (Pmax); center-of-pressure speed (CoP-speed); length surface function (LSF); center-of-pressure sway area (CoPsa); arch index (AI); and foot contact area.

Figure 3 illustrates the outcome of the multiple correlation analysis between pairs of stabilometric and plantar pressure parameters under OE and CE conditions. The *R* ^2^ of the correlation between each pair of parameters are graphically reported in a 31 x 31 matrix, using round markers whose size represents the magnitude of a statistically significant correlation between a parameter on the *x*-axis and a parameter on the *y*-axis. Positive correlations are represented by green round markers and negative correlations by black round markers. The *X*-axis parameters were sorted in ascending order by the number of statistically significant correlations (corrected *α* < 0.005) with other parameters. CoP-speed and Pmean Mf (r) were identified as the most independent parameters in OE, showing no correlation with other parameters. In CE, the most independent parameters were Pmean Mf (r), Pmax Mf (r), and LSF; unlike what was observed in OE, CoP-speed was positively correlated with Pmax Rf (l, r), Pmean Rf (l), Load Rf (l), Pmax Tf (l), and CoPsa. A few correlations (≤ 6) were found in OE and CE for Load Tf (l,r), Pmean Mf (l) and Ff (l, r), Pmax Mf (l) and Ff (l, r), and CoPsa. All other parameters showed a larger number of correlations (>6) under both visual conditions.

### 3.3 Inter-side (left vs. right) comparison of plantar pressure parameters

The distribution of stabilometric and plantar pressure parameters under each visual condition is reported as median and 25% and 75% percentiles in Table 2. Significant differences were found between the left and right sides for all parameters (*p* < 0.005), except for Load Ff, Pmean Tf, Pmean Mf, and Pmax Mf under both visual conditions and for Pmax Rf in OE (Table 2). In particular, significant parameters were larger in the right side under both visual conditions, except for Rf.

**TABLE 2:** Inter-subject median and percentiles (25% and 75%) of plantar pressure and stabilometric parameters; significant differences (*p*-value = = 0.005) between left and right (Wilcoxon paired test) and between open and closed eyes (Wilcoxon signed test). Forefoot (Ff); midfoot (Mf); rearfoot (Rf); mean pressure (Pmean); maximum pressure (Pmax); center-of-pressure speed (CoP-speed); center-of-pressure sway area (CoPsa); length surface function (LSF); arch index (AI); and foot contact area (FCA).

Plantar pressure and stabilometric parameters	Side	Open eyes (OE)		Closed eyes (CE)		
		Median (25%; 75%)	*p*-value inter-sides (right vs. left)	Median (25%; 75%)	*p*-value intra-group (right vs. left)	*p*-value inter-visual condition (OE vs. CE)
Load Tf [%]	Left	46.46 (45.65; 48.70)	**< 0.001**	47.53 (46.15; 49)	**< 0.001**	0.224
	Right	53.54 (51.30; 54.35)		52.48 (51.00; 53.85)		0.224
Load Rf [%]	Left	41.19 (36.53; 44.95)	**0.001**	41.84 (35.83; 45.03)	**0.001**	0.469
	Right	36.95 (33.88; 42.00)		37.44 (34.43; 41.80)		0.420
Load Mf [%]	Left	14.05 (8.58; 25.13)	**0.001**	14.91 (9.05; 26.48)	**< 0.001**	0.419
	Right	20.79 (12.98; 24.95)		21.42 (13.45; 24.70)		0.370
Load Ff [%]	Left	43.91 (41.03; 45.05)	0.133	43.16 (40.35; 44.53)	0.13	0.329
	Right	43.05 (40.85; 43.85)		41.99 (39.20; 44.25)		0.312
Pmean Tf [KPa]	Left	24.04 (22.96; 27.89)	0.393	23.45 (22.27; 26.49)	0.108	0192
	Right	24.39 (22.79; 27.32)		23.54 (21.94; 25.70)		0.101
Pmean Rf [KPa]	Left	27.35 (23.83; 35.58)	**< 0.001**	27 (23.44; 32.43)	**0.004**	0.261
	Right	23.19 (18.70; 31)		22.02 (18.02; 33.17)		0.395
Pmean Mf [KPa]	Left	16.38 (15; 18.75)	0.026	15.88 (15; 18.25)	0.154	0.312
	Right	18.25 (17.00; 20)		17.75 (15.75; 18.50)		0.068
Pmean Ff [KPa]	Left	59.07 (53.15; 67.85)	**< 0.001**	57.66 (47.31; 62.64)	**0.003**	0.212
	Right	69.92 (60.34; 83.98)		59.22 (55.71; 69.20)		0.040
Pmax Tf [KPa]	Left	62.78 (57.32; 71.52)	**0.001**	60.57 (58.09; 68.64)	**< 0.001**	0.335
	Right	58.90 (53.27; 65.87)		57.08 (52.11; 64.55)		0.234
Pmax Rf [KPa]	Left	67.25 (59.50; 80.50)	0.025	64.5 (56.63; 68.13)	**0.002**	0.192
	Right	63.25 (55.75; 71)		58.47 (53.06; 6.06)		0.115
Pmax Mf [KPa]	Left	30.87(27.25; 36.63)	0.053	31.38 (27.50; 37.25)	0.332	0.469
	Right	35.75 (31.75; 37.94)		34 (29.5; 35.7)		0.084
Pmax Ff [KPa]	Left	44.50 (41.75; 49.25)	**< 0.001**	42.50 (41.22; 45.75)	**0.001**	0.352
	Right	49.63 (48.25; 55)		47.47 (45.25; 49.13)		0.011
FCA [mm^2^]	Left	118 (97.31; 134.44)	**< 0.001**	120.25 (100.88; 139.31)	**< 0.001**	0.303
	Right	132.5(112.19; 160.38)		136 (116.88; 164.94)		0.201
AI [%]	Left	14.05 (8.57; 25.27)	**0.002**	14.92 (9.05; 26.48)	**< 0.001**	0.419
	Right	20.63 (12.98; 24.95)		21.43 (13.44; 24.71)		0.370
CoPsa [mm^2^]		28.80 (23.50; 41.69)		24.98 (13.17; 29.22)		0.055
CoP-speed [mm/s]		3.44 (3.13; 3.970)		3.24 (3.00; 3.93)		0.192
LSF [mm^-1^]		4.69 (3.80; 7.10)		7.85 (5.28; 9.68)		0.040

Bold values are highlight statistical significance (*p* < 0.005).

### 3.4 Intra-side comparison of stabilometric and plantar pressure parameters under OE and CE conditions

Table 2 reports the inter-subject median and percentile (25% and 75%) distributions of all parameters under both visual conditions. No significant differences were observed in stabilometric and plantar pressure parameters between OE and CE (p ≤ 0.005). However, a trend for larger LSF and lower CoPsa, Pmean, and Pmax Ff (r) was found in CE.

## 4 Discussion

Postural sway and plantar pressure parameters in the upright standing position can be modulated in response to different visual inputs (Stoffregen et al., 2000; Bellizzi et al., 2011; Nishiike et al., 2013). Understanding the effects of open- and closed-eye conditions on these parameters and on their variability is important for their interpretation in clinical and research investigations.

The first aim of the present study was to analyze the intra-subject variability in stabilometric and plantar pressure parameters in healthy subjects during the bipedal standing test under OE and CE conditions. The coefficient of variation was lower than 20% in CE and OE across all parameters, except for the right arch index in CE (≤40%) and CoPsa and LSF (≥50%) under both visual conditions. These findings are partly in agreement with a review that identified mean pressure, percentage of body weight distribution, and foot contact area as some of the most reliable measures (Hébert-Losier and Murray, 2020). The outcome of the variability analysis under the CE condition is also in agreement with what was observed previously in OE (Figure 2) (Fullin et al., 2022); thus, the variability in stabilometric and plantar pressure parameters does not seem to be affected by the different visual conditions. The only exception is the right arch index, which suggests a greater dynamic role of the dominant foot with respect to the postural adaptations occurring with closed eyes.

To the best of the authors’ knowledge, this study is the first to investigate the correlations between pairs of postural stability and plantar pressure parameters under both visual conditions. In order to better highlight and visually determine the overall outcome of the correlation analysis, a novel graphical approach was established (Figure 3). This visual representation allows for a faster and more comprehensive understanding of which parameters are more independent (i.e., less correlated to other parameters) in the characterization of the phenomenon, especially when several parameters are under investigation. Markers’ dimensions and colors allow us to quickly identify the magnitude and direction of each correlation. According to the outcome of the present study (Figure 3), mean and peak pressures at midfoot and total foot loads (l, r) were among the most independent parameters under both visual conditions, whereas arch index (l, r) and rearfoot loads (l, r) were among the most correlated with other parameters. In particular, CoP-speed and mean pressure at the midfoot were identified as the most independent parameters in OE. Under the CE condition, mean and peak pressures at midfoot (r > l) and LSF were the most independent parameters. Unlike what was observed in OE, CoP-speed in CE was positively correlated with the following variables: Pmax rearfoot (l, r), Pmax total foot (l), Pmean and load rearfoot (l), and CoPsa. These positive correlations, associated with the high reliability of CoP-speed, seem to suggest a different control mechanism of body sway with closed eyes compared to open eyes, underlying a possible functional relationship of CoP-speed with the CoP sway area and rearfoot load in the non-dominant side. The length surface function also showed different correlations between the two visual conditions. As predictable, it was negatively correlated with CoPsa in OE since it represents the ratio between the distance covered by the CoP during a standing trial (CoP-length) and CoPsa, whereas it was independent in CE (i.e., showed no significant correlations with other parameters). This different behavior between open and closed eyes is the opposite of what occurs for CoP-speed and could likely be explained by the mutual relationship of CoP-speed and LSF with CoP-length.

The present study provides a deeper insight into the effects of visual stimuli on stabilometric and plantar pressure parameters, which were previously reported in OE (Fullin et al., 2022). In agreement with what was found in OE (Fullin et al., 2022), significant differences were observed between the dominant (right) and non-dominant (left) sides for several parameters (Table 2). In particular, loads were significantly lower at the rearfoot and significantly larger at the midfoot of the dominant side under both visual conditions. In terms of mean and peak pressures, a significant decrease at the rearfoot and a significant increase at the forefoot were found in the dominant side under both visual conditions. This finding was also associated with a larger foot contact area and a larger percentage of the midfoot contact area (larger AI) in the dominant side for OE and CE (Table 2). According to previous studies (Hennig and Sterzing, 2009; Machado et al., 2015), the midfoot seems to be the most sensitive region to both tactile and vibration stimuli; therefore, it could be hypothesized that the midfoot plays an important role in the balance control mechanism. The larger midfoot contact area found in the dominant side would increase the number of receptors responsible for the spatial orientation of the body center of mass in contact with the ground. This seems to be consistent with the outcome of the correlation analysis (Figure 3), which showed that mean and peak pressures at the midfoot are two of the most independent parameters and thus are good indicators for the characterization of the balance control under both visual conditions.

The visual stimulus affected several parameters (Table 2). In particular, a trend for larger LSF and lower CoPsa and mean and peak pressures at the forefoot in the right side was found in CE with respect to the OE condition (Fullin et al., 2022). Unlike what was reported in other studies (Prieto et al., 1996; Tanaka et al., 2000; Hue et al., 2006; Vieira Tde et al., 2009; Bellizzi et al., 2011; Baldini et al., 2013; Santana Castro et al., 2021; Nishiike et al., 2013; Rodríguez-Rubio et al., 2020; De Blasiis et al., 2021b; Quialheiro et al., 2021; Scoppa and Gallamini, 2021), CoPsa and CoP-speed were not larger in the CE condition. This finding could be probably explained by the effect of the longer visual target distance with respect to the clinical stabilometry standardization (Tarantola et al., 1997; Tanaka et al., 2000; Chiari et al., 2002). Indeed, this variable has been shown to be positively correlated with CoPsa and CoP-speed (Stoffregen et al., 2000).

The limitations to the study were the relatively small sample size, due to the rather strict inclusion criteria, particularly for visual and dental impairments; it should also be noted that while most parameters showed normal distribution, the normality assumption was not guaranteed for all parameters, and this violation may have slightly affected the outcome of the correlation analysis. Moreover, the self-selected feet position may be considered a methodological bias, but the indication to place the feet close but not together in order to obtain a comfortable upright bipedal standing posture may be a good standardization method according to the neurophysiological principle of the natural postural control strategy instead of imposing an unnatural posture. In addition, the position of the visual target (2.8 m away from the subject) may have affected the stabilometric and plantar pressure measurements under the OE condition; in particular, an increase in CoPsa with the increasing target distance has been reported (Stoffregen et al., 2000). The effect of the visual target distance should be investigated in future studies.

The present study aided in establishing the most reliable and independent stabilometric and plantar pressure parameters for the evaluation of bipedal standing posture under open- and closed-eye conditions in a healthy young population. As expected, significant differences were observed between the left and right sides and between correlations under two visual conditions. While more data from a larger population should be sought, the study has highlighted the importance of the dominant side and the specific role of the midfoot in the balance control.

## Data Availability

The raw data supporting the conclusion of this article will be made available by the authors, without undue reservation.
